# Optimizing the Treatment Pattern for *De Novo* Metastatic Nasopharyngeal Carcinoma Patients: A Large-Scale Retrospective Cohort Study

**DOI:** 10.3389/fonc.2020.543646

**Published:** 2020-10-23

**Authors:** Xue-Song Sun, Yu-Jing Liang, Qiu-Yan Chen, Shan-Shan Guo, Li-Ting Liu, Rui Sun, Dong-Hua Luo, Lin-Quan Tang, Hai-Qiang Mai

**Affiliations:** ^1^ State Key Laboratory of Oncology in South China, Sun Yat-sen University Cancer Center, Guangzhou, China; ^2^ Collaborative Innovation Center for Cancer Medicine, Sun Yat-sen University Cancer Center, Guangzhou, China; ^3^ Guangdong Key Laboratory of Nasopharyngeal Carcinoma Diagnosis and Therapy, Sun Yat-sen University Cancer Center, Guangzhou, China; ^4^ Department of Nasopharyngeal Carcinoma, Sun Yat-sen University Cancer Center, Guangzhou, China

**Keywords:** metastatic nasopharyngeal carcinoma, palliative chemotherapy, locoregional radiotherapy, concurrent chemotherapy, overall survival

## Abstract

**Objectives:**

To investigate the optimal treatment pattern in patients with *de novo* metastatic nasopharyngeal carcinoma (NPC).

**Methods:**

We assessed 502 consecutive and unselected *de novo* metastatic NPC patients in Sun Yat-sen University Cancer Center (SYSUCC) from November 2006 to October 2016 in our study. All patients were treated with palliative chemotherapy (PCT) and 308 patients received locoregional radiotherapy (LRRT) subsequently. Our primary study endpoint was overall survival (OS).

**Results:**

The patients treated with LRRT were associated with improved survival on univariate analysis (3-year OS rate 63.7% vs. 31.8%, P < 0.001) and multivariate analysis (HR 0.52, 95%CI 0.40–0.68, P < 0.001). The overall survival benefit of more than 4 PCT cycles was significant in female (HR 0.45, 95% CI 0.24–0.86, P = 0.016) and patients with multiple metastatic sites (HR 0.42, 95% CI 0.26–0.66, P < 0.001). The application of concurrent chemotherapy (CCT) was not associated with better survival among patients receiving LRRT (HR 1.31, 95% CI 0.92–1.86, P = 0.141).

**Conclusion:**

LRRT prolonged survival in *de novo* metastatic NPC. For patients treated with multiple metastatic sites, more than 4 cycles of PCT is necessary. CCT does not improve survival in *de novo* metastatic NPC patients.

## Introduction

Nasopharyngeal carcinoma (NPC) is uncommon in most countries but is an endemic malignancy in Southeastern Asia and South China, especially Guangdong province. In 2018, approximately 129,000 new cases of NPC were reported (1). In addition to specific geographic and ethnic distribution, NPC is distinguished from other head and neck carcinomas by its association with Epstein–Barr virus (EBV) infection, its highly aggressive nature, and predisposition of distant metastases (2). Radiotherapy is the fundamental treatment modality and concurrent chemo-radiotherapy (CCRT) is recommended for locoregional advanced NPC according to the National Comprehensive Cancer Network (NCCN) Guidelines (3, 4). Satisfactory tumor control can be achieved in the early stage of disease and even locoregionally advanced NPC can be controlled due to its highly radiosensitive and chemosensitive nature (5). However, distant metastasis remains a key challenge. It has been reported that up to approximately 15% of NPC patients are diagnosed with *de novo* metastatic cancer before any treatment has begun (6). According to previous studies, the overall survival period for NPC patients with distant metastasis at initial diagnosis varies from months to years (7, 8). Moreover, the therapeutic margin of NPC is extremely narrow and currently there is no standard model for the implementation of this comprehensive treatment model. Based on high-level evidence, cisplatin-based combination palliative chemotherapy plays a significant role in treatment of metastatic NPC patients (9, 10). However, treatment of *de novo* metastatic NPC patients must consider the control of primary tumors, which is different from metastatic NPC after treatment. Studies have been conducted to explore the optimal treatment modality for *de novo* metastatic NPC patients. Recently, several retrospective analyses suggested that additional locoregional radiotherapy (LRRT) could improve survival of these patients in addition to palliative chemotherapy (7, 8, 11). However, most studies have been conducted in the two-dimensional conventional radiotherapy (2D-CRT) era and the patient sample is too small to provide convincing evidence (8, 12, 13). Furthermore, the effect of courses and accumulated doses of palliative chemotherapy (PCT) and concurrent chemotherapy (CCT) on clinical outcomes of *de novo* metastatic nasopharyngeal carcinoma patients is largely unknown. Therefore, we conducted a study to investigate the optimal treatment pattern in patients with *de novo* metastatic nasopharyngeal carcinoma.

## Methods and Materials

### Patients

**Figure 1:**
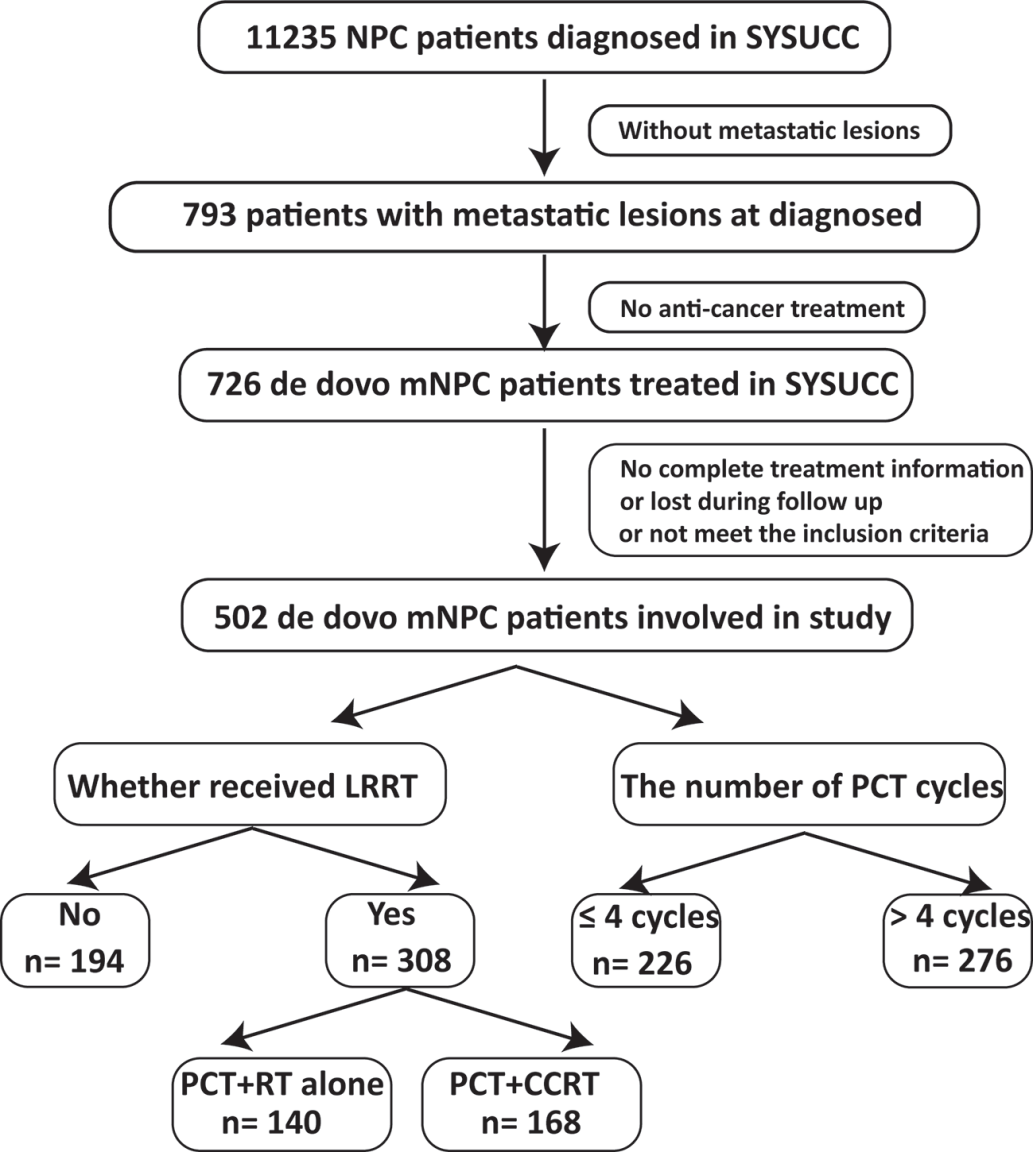
Flow chart of study patient inclusion.

This study is based on the data platform established by Sun Yat Sen University Cancer Center (SYSUCC). From November 2006 to October 2016, a total of 11235 NPC patients were identified. Finally, 502 consecutive and unselected *de novo* metastatic NPC patients were involved in our study. The inclusion criteria included: (1) pathologically confirmed NPC; (2) evidence of distant metastasis confirmed by imaging examinations or pathology; (3) no anti-tumor treatment before metastasis (4) Karnofsky performance score (KPS) >60; (5) adequate organ function; (6) lesions that can be measured radiologically; (7) absence of secondary pregnancy, lactation and other malignant disease. Flow chart of patient inclusion was shown in **Figure 1**. Our study was approved by the Research Ethics Committee of the SYSUCC.

### Diagnosis and Treatment

All patients received a complete pre-treatment assessment at admission and were treated according to the principles of SYSUCC. Detailed information on diagnosis and treatment can be found in **Supplementary Materials**.

### Outcome and Follow-Up

Tumor response was evaluated based on radiological image examinations by 2 experienced radiologists independently according to Response Evaluation Criteria in Solid Tumors criteria (RECIST), version 1.1 (14, 15). The primary endpoint of our study was overall survival (OS), defined as the length of time from the date of diagnosis to the date of death of any cause. Patients who were lost to follow-up or were still alive had their data censored in last follow-up. After systemic therapy, patients were evaluated at least once every 3 months during the first 3 years and thereafter every 6 months until death. Physical examination, nasopharyngoscopy, MRI with contrast of head and neck, CT/MR with contrast of the metastatic sites, abdominal sonography, chest radiography, and plasma EBV DNA measurement were all routinely performed. PET-CT and others were considered if necessary.

### Statistical Analysis

To assess whether there were statistical relationships between clinical characteristics and treatment groups, Pearson χ2 test was used. Survival curves were generated using the Kaplan-Meier method, and survival rates were compared using the log-rank test. Landmark analyses for patients with ≥1 and ≥3 years OS were used to account for potential immortal-time biases (16). Multivariate Cox regression analysis was used to estimate the hazard ratio (HR) and 95% confidence interval (CI) for the correlations between variables and OS. Forest plots were generated to present the results of subgroup analyses for OS and multivariate HR was adjusted for following factors: age, gender, T stage, N stage, metastatic sites, tumor response to PCT and number of PCT cycle, excluding the stratification covariates. All statistical tests were 2-tailed and P < 0.05 was considered as statistical significance. All statistical analyses were conducted using the Statistical Package for Social Sciences (SPSS, Mac version 21.0, Chicago, IL).

## Results

### Clinical Characteristics and Survival 

The median patient age was 47 years (range, 12–77 years); 417 patients (83.1%) were men; 308 patients (61.4%) received LRRT after PCT and 276 (55.0%) patients received more than 4 cycles PCT. **Table 1** lists the characteristics of the 502 patients. The median follow-up time was 26.6 months (range, 1–127 months); 260 patients died during the follow-up period. Among them, 257 patients died of tumor progression and three patients died of other reasons (two patients because of treatment-related toxicities and one patient because of cardiac disease). The 1-, 2-, 3-, and 5-year OS rates were 87.9%, 66.2%, 52.8%, and 38.4%, respectively.

**Table 1:** Clinical characteristics of patients diagnosed with *de novo* metastatic NPC.

Characteristic	Followed by LRRT after PCT			The number of PCT cycles		
****	Yes	No	P value	≤4	>4	P value
Total	308	194		226	276	
**Gender**			1.000			0.283
Male	256(83.1%)	161(83.0%)		183(81.0%)	234(84.8%)	
Female	52(16.9%)	33(17.0%)		43(19.0%)	42(15.2%)	
**Age (yr)**			0.169			0.180
≤47	168(54.5%)	93(47.9%)		110(48.7%)	151(54.7%)	
>47	140(45.5%)	101(52.1%)		116(51.3%)	125(45.3%)	
T stage*			0.423			0.684
T1	11(3.6%)	10(5.2%)		10(4.4%)	11(4.0%)	
T2	44(14.3%)	19(9.8%)		31(13.7%)	32(11.6%)	
T3	149(48.4%)	99(51.0%)		105(46.5%)	143(51.8%)	
T4	104(33.8%)	66(34.0%)		80(35.4%)	90(32.6%)	
N stage*			0.333			0.220
N0	13(4.2%)	3(1.5%)		10(4.4%)	6(2.2%)	
N1	57(18.5%)	32(16.5%)		46(20.4%)	43(15.6%)	
N2	123(39.9%)	79(40.7%)		88(38.9%)	114(41.3%)	
N3	115(37.3%)	80(41.2%)		82(36.3%)	113(40.9%)	
**Metastatic sites**			<0.001			<0.001
Bone	166(53.9%)	66(34.0%)		117(51.8%)	115(41.7%)	
Lung	37(12.0%)	25(12.9%)		28(12.4%)	34(12.3%)	
Liver	23(7.5%)	26(13.4%)		13(5.8%)	36(13.0%)	
Other site	32(10.4%)	2(1.0%)		24(10.6%)	10(3.6%)	
Multiple sites	50(16.2%)	75(38.7%)		44(19.5%)	81(29.3%)	
**Tumor response to PCT**			<0.001			0.051
CR/PR	213(69.2%)	102(52.6%)		131(58.0%)	184(66.7%)	
SD/PD	95(30.8%)	92(47.4%)		95(42.0%)	92(33.3%)	
**Number of PCT cycles**						
≤4	156(50.6%)	70(36.1%)				
>4	152(49.4%)	124(63.9%)				
**LRRT**						0.002
No				70(31.0%)	124(44.9%)	
Yes				156(69.0%)	152(55.1%)	

PCT, palliative chemotherapy; LRRT, locoregional radiotherapy; CR, complete response; PR, partial response; PD, disease progression; SD, stable disease; TPF, cisplatin plus docetaxel plus 5-fluorouracil; TP, cisplatin plus docetaxel; PF, cisplatin plus 5-fluorouracil; GP, cisplatin plus gemcitabine.

*According to the 8th edition of the UICC/AJCC staging system.

P value was calculated with the Pearson χ2 test.

### Relationship Between LRRT, PCT Cycles, and Clinical Outcome

**Figure 2:**
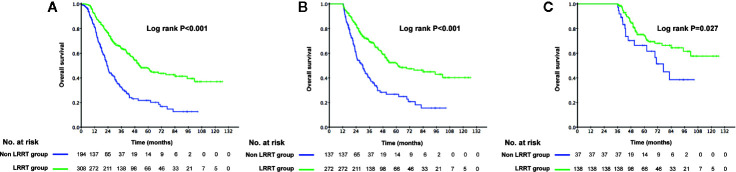
Kaplan–Meier curves of overall survival in 502 de novo metastatic nasopharyngeal carcinoma (NPC) patients treated with locoregional radiotherapy (LRRT) and without LRRT **(A)**, Landmark analyses of overall survival for survivors of ≥1 year **(B)** and ≥3 years **(C)**.

**Figure 3:**
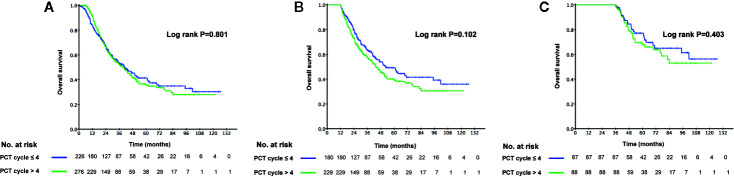
Kaplan–Meier curves of overall survival in 502 de novo metastatic nasopharyngeal carcinoma (NPC) patients accepting ≤4 cycles palliative chemotherapy (PCT) and accepting >4 cycles of PCT **(A)**, Landmark analyses of overall survival for survivors of ≥1 year **(B)** and ≥3 years **(C)**.

The 3-year OS rate in patients treated with LRRT was significantly higher than for patients who did not receive treatment with LRRT (63.7%, 95% confidence interval [CI] 58.0–69.4% versus 31.8%, 95% CI 24.0–39.6%, P < 0.001) (**Figure 2A**). Landmark analyses were used to evaluate the impact of LRRT for survivors over 1 year and 3 years. As displayed in **Figures 2B, C**, LRRT was associated with improved OS at each landmark, the P value for ≥1 and ≥3 years survivors was <0.001 and 0.027 respectively. However, the patients in the different PCT cycle groups were not significantly different (**Figures 3A–C**). All of the following potential prognostic factors were considered in the Cox proportional hazards model: patient age (≤47 years or >47), gender (male or female), T stage (T1-2 or T3-4), N stage (N0-1 or N2-3), metastatic sites (single metastatic site or multiple metastatic sites), number of PCT cycle (≤4 or >4), tumor response to PCT (complete response [CR]/partial response [PR] or stable disease [SD]/disease progression [PD]) and whether PCT was followed by LRRT. **Table 2** demonstrates LRRT were independent prognostic factors in the multivariate model for OS (HR 0.52, 95%CI 0.40–0.68, P < 0.001). N stage, metastatic sites and tumor response to PCT were other independent prognostic factors, whereas the number of PCT cycles did not confer survival benefit (HR 0.79, 95%CI 0.61–1.02, P = 0.075).

**Table 2:** Multivariate analysis for OS.

Characteristic	HR	95%CI	P value
Gender	0.86	0.62–1.20	0.382
Age (year)	1.18	0.92–1.51	0.192
T stage	0.97	0.70–1.34	0.857
N stage	1.68	1.22–2.31	0.001
Metastatic sites	2.84	2.14–3.77	<0.001
Number of PCT cycles	0.79	0.61–1.02	0.075
Tumor response to PCT	1.75	1.35–2.26	<0.001
LRRT	0.52	0.40–0.68	<0.001

CI, confidence interval; HR, hazard ratio; PCT, palliative chemotherapy; LRRT, locoregional radiotherapy.

A Cox proportional hazards regression model was used to detect variables one by one without adjustment. All variables were transformed into categorical variables. HRs were calculated for Gender (Female vs. Male); Age (y) (>47 vs. ≤47); T stage (T3-4 vs. T1-2); N stage (N2-3 vs. N0-1); Metastatic sites (Multiple sites vs. Single site); Number of PCT cycle (>4 vs. ≤4); Tumor response to PCT (SD/PD vs. CR/PR); LRRT (Yes vs. No).

### Subgroup Analyses

**Figure 4:**
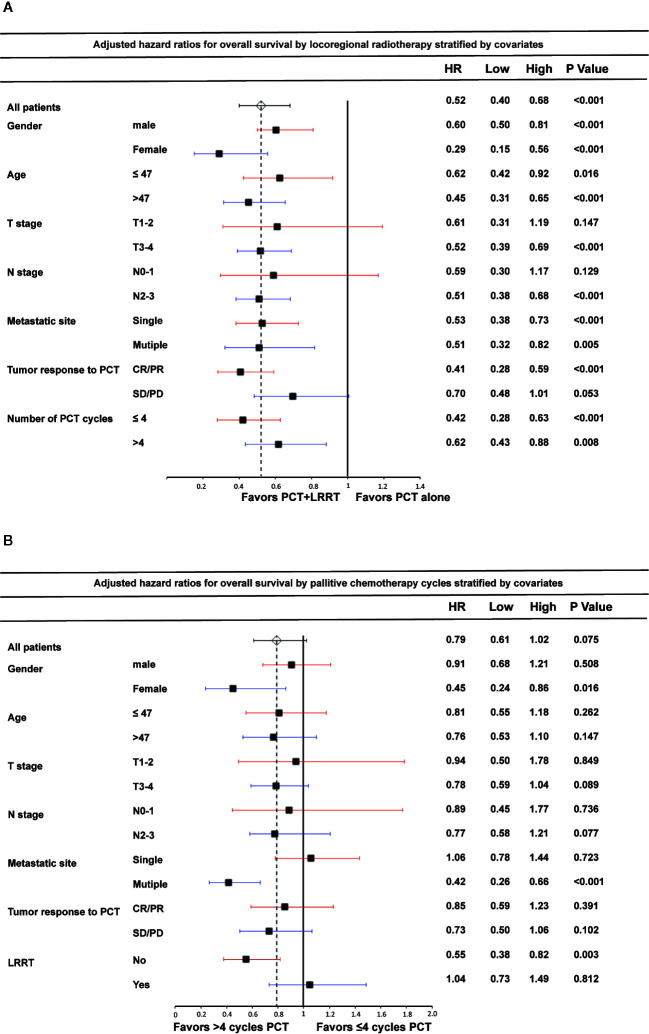
Forest plot of the association between locoregional radiotherapy (LRRT) and overall survival by subgroup **(A)**; palliative chemotherapy (PCT) cycles and overall survival by subgroup **(B)**. Multivariate hazard ratios (HR) displayed are adjusted for the factors described in the methods section. Low and High refer to the lower and upper limit of the 95% confidence interval, respectively.

In the analyses of the association between LRRT and OS by different subgroups, no significant interactions were observed between the effect of LRRT and age, gender, metastatic sites and the number of PCT cycles (**Figure 4A**). Notably, LRRT was not associated with improved OS for patients SD/PD after PCT (HR 0.70, 95%CI 0.48–1.01, P = 0.053). Among patients in T1-2 and N0-1 subgroup, the benefit of LRRT was also not significant. In terms of PCT cycles, more than 4 cycles PCT showed no significant survival differences in subgroups by age, T stage, N stage and tumor response to PCT (**Figure 4B**). However, with restriction to patients with multiple metastatic sites, the effect of more than 4 cycles PCT on OS became significant (HR 0.42, 95%CI 0.26–0.66, P < 0.001). The survival benefit was also shown in separate subgroups of female (HR 0.45, 95%CI 0.24–0.86, P = 0.016) and non-LRRT (HR 0.55, 95%CI 0.38–0.82, P = 0.003).

### The Prognostic Impact of CCT in De Novo Metastatic NPC Patients

**Figure 5:**
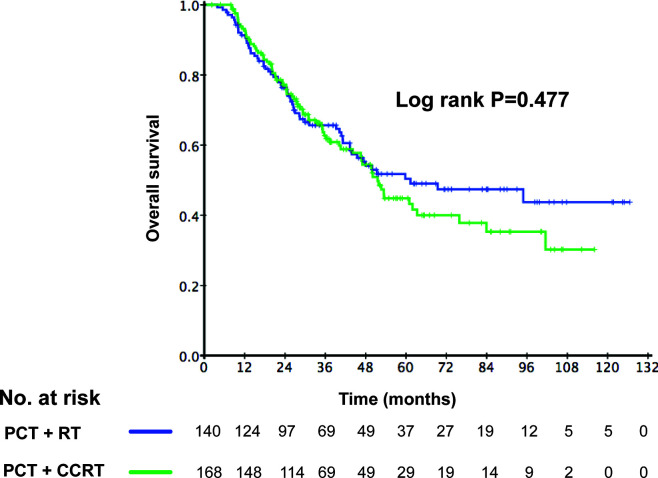
Kaplan–Meier curves of overall survival according to the application of concurrent chemotherapy (CCT) in 308 *de novo* metastatic nasopharyngeal carcinoma (NPC) patients treated with locoregional radiotherapy (LRRT).

Among the 308 *de novo* metastatic NPC patients treated with LRRT, 168 patients received cisplatin-based chemotherapy during radiotherapy. According to the application of CCT, we divided these patients into 2 groups: patients receiving LRRT alone and patients receiving CCRT after PCT. There was no difference in clinical outcome between the two groups. The 3-year OS rates in the LRRT and CCRT groups were 64.6% and 61.8%, respectively (P = 0.477). Kaplan–Meier survival curves are represented in **Figure 5**. In the multivariate analysis, **Table 3** shows that patients receiving CCT was not associated with survival benefit (HR 1.31, 95% CI 0.92–1.86, P = 0.141).

**Table 3:** Multivariate analysis for OS in LRRT group.

Characteristic	HR	95%CI	P value
Gender	0.60	0.35–0.95	0.032
Age (yr)	1.03	0.73–1.44	0.885
T stage	0.97	0.63–1.51	0.897
N stage	1.54	1.00–2.37	0.048
Metastatic sites	2.79	1.84–4.22	<0.001
Number of PCT cycles	1.14	0.78–1.65	0.501
Tumor response to PCT	2.38	1.68–3.37	<0.001
CCT	1.31	0.92-1.86	0.141

CI, confidence interval; HR, hazard ratio; PCT, palliative chemotherapy; LRRT, locoregional radiotherapy; CCT, concurrent chemotherapy.

A Cox proportional hazards regression model was used to detect variables one by one without adjustment. All variables were transformed into categorical variables. HRs were calculated for Gender (Female vs. Male); Age (y) (>47 vs. ≤47); T stage (T3-4 vs. T1-2); N stage (N2-3 vs. N0-1); Metastatic sites (Multiple sites vs. Single site); Number of PCT cycle (>4 vs. ≤4); Tumor response to PCT (SD/PD vs. CR/PR); CCT (Yes vs. No).

## Discussion

Distant metastasis has been a leading cause of death in NPC patients (17, 18). The best treatment strategy for these patients is still under discussion (19–21). The benefits of PCT have been demonstrated in previous studies and this treatment regimen is considered as the only possibly curative option. A platinum-based combination regimen is the most widely used regimen with objective response rates of 55%–80% (22–24). Radiotherapy, especially IMRT, has become the main treatment method for non-metastatic NPC (25–28). However, the value of LRRT in patients with *de novo* metastatic NPC has not been clearly evaluated. In our study, we retrospectively compared the clinical outcome of *de novo* NPC patients treated with or without LRRT and further investigated the prognostic impact of accumulated doses of chemotherapy.

With the development of RT technology and the application of platinum combination therapy, more and more studies showed that LRRT could prolong the survival time of *de novo* metastatic NPC patients (8, 13, 21). In a retrospective study of 125 NPC patients with initial metastases, Yeh et al. reported that the 2-year OS rate was 24.0% in patients receiving radiotherapy, whereas it was only 10% in those who received chemotherapy alone (21). Another study by Lu et al. retrospectively analyzed 234 patients and found that LRRT significantly extended patient OS compared with those treated with chemotherapy alone, with the 3-year OS rate increasing from 12.4% to 48.3% (13). Similar results were also obtained in a study by Chen et al., which retrospectively evaluated the impact of different treatment strategies on patient survival (8). Although these aforementioned studies demonstrated a survival advantage of LRRT, the predominant radiation technique used was conventional 2D-CRT. In our study, the main application of radiotherapy technology was IMRT (229 of 308 patients received LRRT). Different from non-metastatic NPC patients, the correlation between TN staging and treatment method was weak, which could be confirmed from previous studies (8, 29). The 3-year OS rate for patients receiving LRRT after chemotherapy was as high as 63.7%, which was significantly higher than those receiving PCT alone at only 31.8% (P < 0.001). LRRT prevented patients from experiencing local failure such as bleeding, visual and hearing impairment, severe headache and cranial nerve paralyses. More importantly, it is believed that LRRT is a potent method of removing the primary tumor, which is a good way of preventing further metastatic progression from the primary “source” of tumor. However, for patients who are chemotherapy insensitive or who have distant lesions that are difficult to eliminate (SD/PD after PCT), the benefit of LRRT was not significant. For these patients, the distant lesions were not under control and the LRRT was unable to control the distant lesions. Previous studies also verified that LRRT does not confer benefit to *de novo* metastatic NPC patients with liver metastases or who have had PD after receiving PCT (11, 13).

Systemic chemotherapy has been established as the standard treatment method in *de novo* metastatic NPC. Nevertheless, the optimal number of PCT cycles was still uncertain and several previous studies have obtained different results. Fandi et al. reported a retrospective study involving 20 metastatic NPC and showed that six cycles PCT was necessary (19). Similarly, Lu et al. proved that patients receiving at least four cycles of chemotherapy had a significantly better survival outcome than those receiving less than four cycles (13). However, in another study, there was no significant survival benefit for patients receiving at least four cycles of chemotherapy versus those receiving less than 4 cycles (7, 8). Hu et al. also state that an increasing number of chemotherapy cycles might precipitate acquired chemo-resistance (30). In the current study, the cut-off value for PCT cycles was four according to the median number of cycles in our cohort. Among all the patients, there was no survival benefit in patients receiving more than 4 cycles of PCT compared with patients receiving up to and including 4 cycles. In the subgroup analyses, we identified a portion of patients benefiting from more PCT cycles. In multiple metastatic sites subgroup, patients who received more than four cycles PCT had significantly better OS compared to the patients who did not, suggesting that higher dose chemotherapy is necessary to control distant lesions and subclinical lesions for this kind of patients. Interestingly, more then 4 PCT cycles were also associated with improved OS in non-LRRT group. As mentioned above, LRRT is an intensive treatment method, which is a potent method of both controlling primary lesions and preventing metastatic lesion progression from the original focus. This further reduces the tumor burden. Thus, the dose of PCT can reduce among patients when it is followed by LRRT. The ratio of patients with multiple metastatic sites was higher in the non-LRRT group, which may be another reason. Additionally, higher PCT does benefit the female patients, suggesting that female were more appropriate for an intensive treatment method.

CCRT has been established as the standard of treatment for advanced non-metastatic locoregional advanced NPC (3, 4). In previous study, our group further proved that cumulative doses of cisplatin in CCT is also significantly associated with OS and DMFS in these patients (31). However, rare study investigated the value of CCT in metastatic NPC patients. In our study, we divided the *de novo* metastatic patients followed by LRRT after PCT into two groups according to the application of CCT. Inconsistent with results from non-metastatic advanced NPC, there was no significant difference between the patients in the LRRT and CCRT. Besides, side effects of concurrent chemotherapy such as nausea, emesis, and anesthesia are hard for patients to endure after undergoing several cycles of PCT before LRRT. Thus, the application of CCT is not recommended according to the result of our study.

There are several limitations to this study. First, this is a retrospective study and the selection bias and potential imbalances in other variables was inevitable. Secondary, the EBV DNA, which was an important biomarker for NPC, was not involved in our study. The third limitation is that the median follow-up duration was 26.6 months and longer follow-up time is needed to prove our results. Finally, the data were obtained from one center and the results should be validated by a multi-centric clinical study.

## Conclusion 

The use of LRRT following systemic PCT prolonged survival in patients with *de novo* metastatic NPC and should be considered as a first-line treatment method. For patients who have multiple metastatic sites, higher doses of PCT (more than 4 cycles) are necessary. CCT is not associated with significantly better survival, so it is dispensable to be given during LRRT in *de novo* metastatic NPC patients.

## Data Availability Statement

All datasets generated for this study are included in the article/**Supplementary Material**.

## Ethics Statement

This retrospective study was approved by the Clinical Research Committee of Sun Yat Sen University Cancer Center. Patients were required to provide written informed consent before enrolling in the study. The patients/participants provided their written informed consent to participate in this study.

## Author Contributions

Study concepts: H-QM, L-QT, Q-YC. Study design: X-SS, Y-JL, Q-YC. Data collection: X-SS, Y-JL, S-SG, L-TL, RS, D-HL. Quality control of data and algorithms: X-SS, Y-JL, Q-YC. Data analysis and interpretation: X-SS, Y-JL, Q-YC. Statistical analysis: X-SS, Y-JL, Q-YC. Manuscript preparation: X-SS, Y-JL, S-SG, L-TL, RS, D-HL. Manuscript editing: X-SS, Y-JL, Q-YC. Manuscript review: X-SS, Y-JL, S-SG, L-TL, RS, D-HL, H-QM, L-QT, Q-YC. All authors contributed to the article and approved the submitted version.

## Funding

This work was supported by grants from the National Key R&D Program of China (2017YFC0908500, 2017YFC1309003), the National Natural Science Foundation of China (No. 81425018, No. 81672868, No.81802775), the Sci-Tech Project Foundation of Guangzhou City (201707020039), the Sun Yat-sen University Clinical Research 5010 Program, the Special Support Plan of Guangdong Province (No. 2014TX01R145), the Natural Science Foundation of Guangdong Province (No.2017A030312003, No.2018A0303131004), the Natural Science Foundation of Guangdong Province for Distinguished Young Scholar(No. 2018B030306001), the Sci-Tech Project Foundation of Guangdong Province (No. 2014A020212103), the Health & Medical Collaborative Innovation Project of Guangzhou City (No. 201400000001, No.201803040003), Pearl River S&T Nova Program of Guangzhou (No. 201806010135), the Planned Science and Technology Project of Guangdong Province (2019B020230002), the National Science & Technology Pillar Program during the Twelfth Five-year Plan Period (No. 2014BAI09B10), and the Fundamental Research Funds for the Central Universities.

## Conflict of Interest

The authors declare that the research was conducted in the absence of any commercial or financial relationships that could be construed as a potential conflict of interest.
