# Supplementary material for: Optimizing the Treatment Pattern for De Novo Metastatic Nasopharyngeal Carcinoma Patients: A Large-Scale Retrospective Cohort Study
Source: Front Oncol. 2020 Oct 23;10:543646. doi: 10.3389/fonc.2020.543646 (PMC7645036; doi:10.3389/fonc.2020.543646)
Supplement: Supplementary file 1 [file DataSheet_1.docx]

***Diagnosis and treatment***

A series of routine evaluations were conducted before treatment, including physical examination, head and neck magnetic resonance imaging (MRI) with contrast, nasopharyngoscopy, chest radiograph/computed tomography (CT) with contrast, abdominal sonography/CT with contrast, electrocardiography, and bone scans. The positron emission tomography computed tomography (PET-CT) was an alternative diagnostic modality. Further examinations were selected based on the location of the metastatic sites. All eligible patients received cisplatin-based combination PCT. Common chemotherapy regimens include PF: cisplatin (20-30 mg/m^2^ intravenously [IV] on days 1-3) combined with 5-fluorouracil (800-1000 mg/m^2^ continuous IV infusion for 24 hours on days 1-5), GP: cisplatin (20-30 mg/m^2^ [IV] on days 1-3) with gemcitabine (800-1000 mg/m^2^ IV on day 1 and day 8), TP: docetaxel (75 mg/m^2^ IV on day 1) plus cisplatin (20-25 mg/m^2^ IV on days 1-3), TPF: docetaxel (60 mg/m^2^ IV on day 1) plus cisplatin (20-25 mg/m^2^ IV on days 1-3) plus 5-fluorouracil (500-800 mg/m^2^, continuous IV infusion for 24 hours, on days 1-5). The chemotherapeutic agent was administered intravenously every 3 weeks. After PCT, 308 patients were followed by LRRT using Intensity Modulated Radiation Therapy (IMRT) or two-dimensional conventional radiotherapy (2D-CRT). 168 patients received cisplatin-based chemotherapy during radiotherapy. Concurrent chemotherapy was administered tri-weekly cisplatin/nedaplatin (60–100mg/m2) or weekly cisplatin/ nedaplatin (25-35 mg/m2) based regimen for 2 cycles during radiotherapy. A total dose of 66-70 Gy at approximately 2 Gy per fraction was prescribed as 5 daily fractions per week.
